# Identification of novel resistance-associated mutations and discrimination within whole-genome sequences of fluoroquinolone-resistant *Mycobacterium tuberculosis* isolates

**DOI:** 10.1128/spectrum.03930-23

**Published:** 2024-04-30

**Authors:** Yingzhi Chong, Xueying Li, Yifei Long, Shengfei Pei, Qi Ren, Fumin Feng, Haibo Zhang

**Affiliations:** 1 Hebei Coordinated Innovation Center of Occupational Health and Safety, School of Public Health, North China University of Science and Technology, Tangshan, Hebei Province, China; 2 School of Public Health, Shandong Second Medical University, Weifang, Shangdong Province, China; 3 Keenan Research Centre for Biomedical Science, St. Michael’s Hospital, Unity Health Toronto, Toronto, Ontario, Canada; Institut National de Santé Publique du Québec, Sainte-Anne-de-Bellevue, Québec, Canada

**Keywords:** tuberculosis, *Mycobacterium tuberculosis*, fluoroquinolone, resistance, whole-genome sequencing

## Abstract

This study aims to elucidate additional mutation loci associated with fluoroquinolone (FQ) resistance and evaluate the discriminatory capacity of mutation loci and allele mutation frequencies in identifying FQ-resistant *Mycobacterium tuberculosis* (MTB) isolates. A random selection of isolates was extracted from an ongoing collection. Drug resistance was determined using the resazurin microtiter assay (REMA) as the gold standard. Mutation loci and the burden of mutations in the quinolone resistance-determining region (QRDR) were elucidated through whole-genome sequencing (WGS). Novel amino acid mutations, namely, G520D and G520T, were identified in the gyrB and associated with FQ resistance. In the context of distinguishing FQ-resistant isolates, the AUC for the QRDR mutation frequency burden (0.969) surpassed that of the mutation locus (0.929), and this difference was statistically significant (*P* = 0.03). Furthermore, using the resistance mutation locus as a reference, setting the QRDR mutation frequency burden threshold at 1.31% resulted in a 3.60% increase in the accuracy of classifying FQ-resistant isolates (NRI = 3.60%, *P* < 0.001). The QRDR mutation frequency burden appears to offer superior diagnostic efficacy in discriminating FQ-resistant isolates compared to qualitative detection of mutant loci.

**IMPORTANCE:**

Fluoroquinolone (FQ) drugs are recommended as second-line drugs for the treatment of multidrug-resistant tuberculosis. With the massive use of FQ drugs in the clinical treatment of tuberculosis (TB), there is an increasing rate of drug resistance to FQ drugs. In this study, we identified and demonstrated novel amino acid mutations associated with FQ resistance in *Mycobacterium tuberculosis* (MTB), and we quantified the mutation sites and identified the quinolone resistance-determining region (QRDR) mutation frequency burden as a novel diagnostic method for FQ resistance. We hope that the results of this study will provide data support and a theoretical basis for the rapid diagnosis of FQ-resistant MTB.

## INTRODUCTION

Tuberculosis (TB) is a highly contagious disease primarily caused by *Mycobacterium tuberculosis* (MTB), with the lungs being the primary site of infection (1, 2). The global mission to achieve "End TB" is ongoing, yet the diagnosis and treatment of drug-resistant tuberculosis, particularly multidrug-resistant tuberculosis (MDR-TB), continue to present formidable challenges. Among the second-line drugs recommended for MDR-TB treatment in current management programs and ongoing clinical trials, fluoroquinolones (FQs) are of paramount importance (3, 4). Despite their broad-spectrum activity and potent bactericidal properties, FQs are plagued by a high incidence of drug resistance (4, 5). Therefore, the development of a rapid and reliable molecular drug susceptibility testing method is imperative for optimizing the use of FQs in TB treatment.

The mechanism of action of FQs involves binding to DNA gyrase and inhibiting the negative supercoiling of replicated DNA. The most prevalent mechanism of resistance to FQs is attributed to gene mutations occurring in gyrA (Rv0006) and gyrB (Rv0005), the genes encoding the subunits of DNA gyrase (6, 7). Commercialized rapid genetic testing methods, such as line probe assays, can detect approximately 85% of FQ-resistant isolates, whereas conventional PCR or first-generation sequencing methods can only detect around 75% of FQ-resistant isolates (8–10). The World Health Organization (WHO) has endorsed the use of GenoType MTBDRsl VER 2.0 as a rapid diagnostic tool for detecting second-line drug sensitivity in MTB. Presently, it exhibits relatively high efficacy in identifying FQ drug resistance. However, the body of evidence supporting its use in diagnosing FQ resistance remains limited within the realm of evidence-based medicine. Furthermore, the intensive use of FQ drugs exerts significant selective pressure on bacterial strains, potentially leading to the emergence of novel mutations.

Initially, attention was primarily focused on the gyrA gene and its associated mutation sites in the context of FQ resistance. However, as an increasing number of clinical isolates are collected and scrutinized in the laboratory, there is a gradual shift toward investigating mutation sites within the gyrB gene and their relationship with FQ resistance. The quinolone resistance-determining region (QRDR) traditionally encompasses codons 74–113 in gyrA and 461–499 in gyrB, as per the P0C5C5|1–675 numbering scheme (11). Recent studies have unveiled FQ-resistance–conferring mutations occurring just outside the conventional QRDR of gyrB (12). These discoveries necessitate a redefinition of the QRDR in gyrB, potentially extending it from position 461 to 540 (12–14). These findings underscore the importance of acquiring additional data to enhance our comprehension of mutation sites linked to FQ resistance. To this end, the WHO recommends the utilization of whole-genome sequencing (WGS) or next-generation sequencing (NGS) on MTB to identify additional FQ resistance-associated sites and address the current diagnostic challenges.

High-throughput sequencing techniques have significantly bolstered the diagnostic accuracy of drug-resistant MTB by leveraging increased sequencing depth to identify a greater number of drug-resistant mutation sites and employing diverse diagnostic approaches. In addition to evaluating drug resistance based on mutation sites, WGS technology offers the advantage of detecting allelic variant mutation frequencies. Recent support for this concept comes from Fernanda Maruri *et al*., who conducted an analysis of dominant allelic variant mutation frequencies using WGS. Their findings demonstrated that allelic variant mutation frequencies exhibited superior discriminatory power in identifying FQ-resistant isolates compared to qualitative mutation sites (13). However, the effectiveness of allelic variant-based diagnosis of FQ-resistant MTB may be influenced by factors such as the source of the specimen and the genotype of the MTB strain. Consequently, further research is warranted to validate the potential value of allelic variant mutation frequencies in diagnosing FQ-resistant MTB.

In this study, our primary objective was to identify additional FQ-resistant-associated sites through comprehensive whole-genome sequencing. We investigated two distinct types of mutations, namely, mutation sites and allelic variant mutation frequencies, to assess their diagnostic utility in identifying FQ-resistant MTB. By doing so, we aimed to overcome the limitations of current diagnostic methods for FQ-resistant MTB and provide approaches in this critical area of TB management.

## MATERIALS AND METHODS

### Study population

The study was conducted over a period spanning from June 2020 to August 2021, at the Infectious Disease Hospital located in Shijiazhuang City, which is affiliated with the Fifth Hospital of Shijiazhuang City. The study focused on confirmed pulmonary TB patients. Individuals who either expressed unwillingness or were unable to participate in the investigation, patients diagnosed with non-tuberculous mycobacterial lung diseases, and tuberculosis patients with extrapulmonary infections were excluded from the study.

Prior to their inclusion, all participants received comprehensive information regarding the study’s objectives, the utilization of bacterial strains, and potential associated risks. It was explicitly communicated that their regular diagnostic and treatment procedures would remain uninterrupted throughout the entire investigation and that stringent measures would be taken to ensure the confidentiality of their personal information. Informed consent forms were diligently signed by all participants who were enrolled in the study.

Laboratory testing, specimen collection, and their subsequent utilization in this study were subject to approval by the Medical Ethics Committee of North China University of Science and Technology (Approval No: 2021027). All procedures undertaken in this study adhered strictly to the principles outlined in the Helsinki Declaration.

### MTB isolate resistance testing

The MTB isolates were obtained from the Lowenstein–Jensen drug susceptibility test control tubes as part of the clinical assessment of pulmonary TB patients. Following reculturing on the MiddleBrook 7H10 agar medium, isolates from the same culture plate were simultaneously utilized for DNA extraction and the resazurin microtiter assay (REMA).

To assess resistance to FQs, we employed the minimum inhibitory concentration (MIC) as the gold standard. Briefly, a 200-µL aliquot of a 1 McFarland bacterial suspension was inoculated into 20 mL of the MiddleBrook 7H9 liquid broth. Subsequently, 100 µL of this dilution was added to each well of a 96-well plate containing a gradient of drug concentrations. The plates were then incubated in a 37°C environment for 7 days. Following the incubation period, 30 µL of a 0.1 g/L resazurin indicator solution was added to each well, and incubation was continued for an additional 24 hours to evaluate color development.

The MIC was defined as the lowest drug concentration at which the color transitioned from blue to pink. In this study, levofloxacin (Lfx) was used as the representative drug for FQ drugs. The critical concentration (CC) for Lfx was set at 1.0 mg/L. If the MIC value of an isolate exceeded the critical concentration, it was classified as resistant. To ensure the quality of our testing, the H37Rv strain was employed as a positive control strain throughout the experiment.

### DNA extraction and whole-genome sequencing

The extraction of DNA was carried out using the E.Z.N.A. Bacterial DNA Kit, following the manufacturer’s instructions. Prior to whole-genome sequencing, the extracted DNA underwent a series of assessments, including evaluation of DNA purity using a NanoDrop 2000 spectrophotometer, determination of DNA concentration via a Qubit 4.0 fluorescence quantitator, and assessment of DNA integrity through agarose electrophoresis. For inclusion in the genome sequencing analysis, all samples were required to meet the concentration criteria of ≥10 ng/µL and a minimum single reaction sample volume of ≥1 µg.

WGS was performed using the Illumina NovaSeq 6000 sequencer, capable of achieving sequencing depths of up to 800 reads. The sequencing process employed parallel mixed sequencing of multiple samples. Considering the sequencing characteristics of the Illumina platform, paired-end sequencing data were utilized for initial evaluation of the raw sequencing data. This assessment encompassed the examination of quality values (represented as Q scores) and the distribution of GC content. Subsequently, low-quality reads and sequences were removed to obtain clean reads. The sequenced fragments were aligned back to the reference genome using the BWA software, with the Picard software employed to eliminate sequences generated by PCR duplication. The reference genome selected for this analysis was NC_000962.3 *Mycobacterium tuberculosis* H37Rv, sourced from the NCBI database. Finally, the mutation sites were annotated using the ANNOVAR software, following the guidelines presented in the "Catalogue of mutations in Mycobacterium tuberculosis complex and their association with drug resistance" (https://www.who.int/publications/i/item/9789240028173). Sequencing reads obtained from Illumina for WGS (BioProject no. PRJNA1080043) have been deposited in the NCBI server.

### Spoligotyping typing

Spoligotyping typing was conducted on all MTB isolates using the TB-profiler software, utilizing sequences acquired from WGS.

### Innovative mutational loci resistance testing

*Mycobacterium smegmatis* (MS) mc2155, known for its substantial genomic homology with MTB, served as the surrogate organism for MTB in this study. The target gene, gyrB, was ligated individually to the pMV361 plasmid. Subsequently, these plasmids were introduced into the mc2155 strain through electroporation to enable recombinant expression. Control groups were established with recombinant strains overexpressing the wild-type gyrB gene (named pMV361-gyrB). The connection between specific mutation sites and the resulting phenotypic drug susceptibility of these strains was assessed using the resazurin microtiter assay (REMA). For this analysis, a drug concentration gradient was set for Lfx that ranged from 0.015 to 16 mg/L.

The DNA template for these experiments was derived from the H37Rv standard strain, with a DNA concentration of approximately 300 ng/µL. The primer sequences employed are detailed in Table 1. The optimal reaction conditions for amplifying the gyrB gene were determined through gradient PCR as follows: initial denaturation at 94°C for 2 minutes; denaturation at 94°C for 25 seconds, annealing at 52°C for 25 seconds, and extension at 68°C for 5 minutes, for a total of 35 cycles; followed by a final extension at 68°C for 5 minutes. The connection between the gyrB gene and the pMV361 plasmid was established using the DNA Ligation Kit (TaKaRa), as illustrated in Fig. S1. Additional agarose gel electrophoresis images depicting gyrB gene amplification and pMV361-gyrB plasmid construction can be found in Fig. S2. To introduce the mutation sites, the KOD-Plus-Mutagenesis Kit was employed in accordance with the provided instructions (primer sequences listed in Table 1). Further documentation of agarose gel electrophoresis and sequencing images pertaining to pMV361-gyrB-G512R, pMV361-gyrB-G520D, and pMV361-gyrB-G520T plasmids can be found in Fig. S3 and S4.

**TABLE 1:** Primer sequences

Name	Primer sequences 5'–3'	Endonuclease
Wild-type gyrB gene amplification primers		
gyrB-wild-F	ccgg-CAATTG-gtggctgcccagaaaaagaaggccc	Mfe I
gyrB-wild-R	ccgg-AAGCTT-ttagacatccaggaaccgaacatcc	HindIII
Reverse point mutagenesis primers		
gyrB-G520T-F	accaagctgcgctaccacaagatcgtgctg	
gyrB-G520T-R	gatatcgaactcgtcgtggatcccggtgcc	
gyrB-G520D-F	gacaagctgcgctaccacaagatcgtgctg	
gyrB-G520D-R	gatatcgaactcgtcgtggatcccggtgcc	
gyrB-G512R-F	aggatccacgacgagttcgatatcggcaag	
gyrB-G512R-R	ggtgcccagcgccgtgatgatcgcctgaac	

### QRDR and allele-level analysis

Traditionally, the QRDR consists of codons 74–113 in gyrA and 461–499 in gyrB, using the P0C5C5|1–675 numbering scheme as the reference. However, in our study, we extended the QRDR of the gyrB gene based on recent literature findings. According to these reports, the QRDR is now considered to encompass codons 74–113 in gyrA and 461–540 in gyrB (12–14).

### Definition and analysis of allele mutation frequencies, as per a previous study

To analyze allele mutation frequencies, we referred to a previous study. The mutational burden within the QRDR of the gyrA gene was quantified by summing all variant allele frequencies within the gyrA gene’s QRDR loci. A similar procedure was applied to the QRDR loci in gyrB to calculate the mutational burden within the gyrB QRDR (13). Consequently, the cumulative QRDR mutational burden (referred to as QRDR mutation frequency burden) was calculated as the sum of variant allele frequencies within the QRDR regions of both gyrA and gyrB. The overall mutational burden within the target genes was determined as the sum of variant allele frequencies in gyrA/B.

### Statistical analysis

An Excel database was established for data management, and statistical analyses were conducted using SPSS 23.0 and R software. Non-normally distributed continuous data were presented as median (*P_25_* and *P_75_*), and the differences between two groups were compared using the Mann–Whitney U test. The validity (accuracy) of the diagnosis was assessed using sensitivity, specificity, diagnostic odds ratio (DOR), and Youden’s index. The reliability (consistency) of the diagnosis was evaluated using the agreement rate and kappa value. The predictive value was assessed through the positive predictive value (PPV) and negative predictive value (NPV). These validity, reliability, and predictive value metrics collectively assessed the diagnostic performance of drug resistance in clinical MTB isolates. The discriminative ability of diagnostic indicators was evaluated using the area under the ROC curve (AUC). Pairwise comparisons of the AUC were conducted using DeLong’s test, and diagnostic indicator calibration was assessed using the net reclassification index (NRI). Two-sided tests with a significance level of α = 0.05 were employed for statistical analyses.

## RESULTS

### Basic characteristics of DNA gyrase mutations

In this study, we enrolled a total of 139 MTB isolates, comprising 52 FQ-resistant and 87 FQ-sensitive isolates, all derived from 139 patients diagnosed with pulmonary TB. Table 2 provides an overview of all nonsynonymous mutations found within the QRDR of the gyrA/B gene by WGS in the study isolates (nonsynonymous mutations outside the QRDR are shown in Table S1). Notably, with the exception of the S447F mutation, all other nine mutation loci associated with FQ resistance were situated within the DNA gyrase QRDR. According to the WHO catalog of resistance-associated mutations, a total of 46 isolates were identified as harboring 10 distinct resistant mutation loci, with four isolates featuring simultaneous mutations at two loci. Predominantly observed mutation types within the gyrA gene included G88C, G88A, A90V, S91P, D94H, D94Y, D94A, and D94G, while within the gyrB gene, N499D and S447F were frequent. However, these observations represent a subset of the actual number of FQ-resistant isolates, as we also identified amino acid changes at S95T, G512R, G520D, and G520T within the DNA gyrase QRDR (specific information on novel amino acid mutations within the QRDR can be found in Table S2).

**TABLE 2:** All nonsynonymous mutations at QRDR in DNA gyrase in our study population of MTB isolates

Name	Gene	Codon change	Amino acid change	Agreement rate (n, %)
gyrA	Rv0006	Ggc >Tgc	G88C^*a*^	1 (100.00)
gyrA	Rv0006	gGc >gCc	G88A^*a*^	1 (100.00)
gyrA	Rv0006	gCg >gTg	A90V^*a*^	7 (85.71)
gyrA	Rv0006	Tcg > Ccg	S91P^*a*^	2 (100.00)
gyrA	Rv0006	Gac >Cac	D94H^*a*^	1 (100.00)
gyrA	Rv0006	gAc >gCc	D94A^*a*^	11 (100.00)
gyrA	Rv0006	gAc >gGc	D94G^*a*^	17 (94.12)
gyrA	Rv0006	aGc >aCc	S95T	102 (48.04)
gyrB	Rv0005	Aac > Gac	N499D^*a*^	1 (100.00)
gyrB	Rv0005	Ggg >Agg	G512R^*b*^	4 (100.00)
gyrB	Rv0005	gGc >gAc	G520D^*c*^	1 (0.00)
gyrB	Rv0005	GGc >ACc	G520T^*c*^	4 (100.00)
gyrA	Rv0006	Tcg > Ccg gAc >gGc	S91P/D94G^*a*^	1 (100.00)
gyrA	Rv0006	gCg >gTg gAc >gGc	A90V/D94G^*a*^	1 (100.00)
gyrA	Rv0006	gAc >gCc gAc >gGc	D94A/D94G^*a*^	1 (100.00)
gyrA	Rv0006	Gac >Tac gCg >gTg	D94Y/A90V^*a*^	1 (100.00)

^*a*^ The mutation has been associated with ﬂuoroquinolone resistance.

^*b*^ The mutation was only statistically proven to be associated with FQ resistance.

^*c*^ The mutation has not been found to be reported.

### Comparative efficacy of existing resistance mutation loci and mutation frequencies in diagnosing FQ-resistant isolates

The sensitivity for diagnosing FQ-resistant isolates based on the presence of the 10 FQ resistance mutation loci in this study was 82.69%, and a specificity of 96.51%. Analysis of WGS results for all isolates revealed high allele mutation levels at specific mutation loci, with only two FQ-sensitive isolates displaying low-frequency mutations. Consequently, our study focused on assessing the correlation between mutation frequency burden in the QRDR and the entire target gene and FQ resistance.

The mutation frequency burden of each clinical isolate is presented in Table S3. Tables 3 and 4 demonstrate the congruence between mutation frequency burden and mutation loci, as well as the FQ phenotype. Intriguingly, even after excluding isolates with known resistance mutations, both the QRDR mutation frequency burden and the target gene mutation frequency burden in the FQ-resistant group remained significantly higher than those in the FQ-sensitive group, with statistical significance (*P* = 0.001 and *P* = 0.005, respectively). Detailed results are provided in Table 5.

**TABLE 3:** Distribution characteristics of drug-resistant mutant loci and allele mutation frequency burden^*a*^

Type of mutation frequency burden	No drug-resistant loci mutation (*n* = 93)	Drug-resistant loci mutation (*n* = 46)	*Z* value	*P* value
QRDR mutation frequency burden	1.00 (0.00–1.00)	2.00 (1.98–2.00)	−8.43	<0.001
DNA gyrase mutation frequency burden	2.00 (0.00–2.00)	3.00 (2.98–4.00)	−6.60	<0.001

^*a*^ M (*P_25_*, *P_75_*) was used to describe the non-normally distributed measures.

**TABLE 4:** Association of allelic mutation frequency burden with FQ resistance^*a*^

Type of mutation frequency burden	Phenotype sensitivity (*n* = 87)	Phenotypic resistance (*n* = 52)	Z value	*P* value
QRDR mutation frequency burden	1.00 (0.00–1.00)	2.00 (1.97–2.00)	−9.51	<0.001
DNA gyrase mutation frequency burden	1.99 (0.00–2.00)	3.00 (2.98–4.00)	−7.48	<0.001

^*a*^ M (*P_25_*, *P_75_*) was used to describe the non-normally distributed measures.

**TABLE 5:** Distribution characteristics of allelic mutation frequency burden in isolates without drug resistance-associated mutation loci^*a*^

Type of mutation frequency burden	Phenotype sensitivity (*n* = 84)	Phenotypic resistance (*n* = 9)	*Z* value	*P* value
QRDR mutation frequency burden	1.00 (0.00–1.00)	1.98 (1.00–2.00)	−3.45	0.001
DNA gyrase mutation frequency burden	1.99 (0.00–2.00)	3.00 (2.00–24.95)	−2.81	0.005

^*a*^ M (*P_25_*, *P_75_*) was used to describe the non-normally distributed measures.

### Comparison of discriminative ability between mutation loci and mutation frequency burden for identifying FQ-resistant isolates

**Fig 1:**
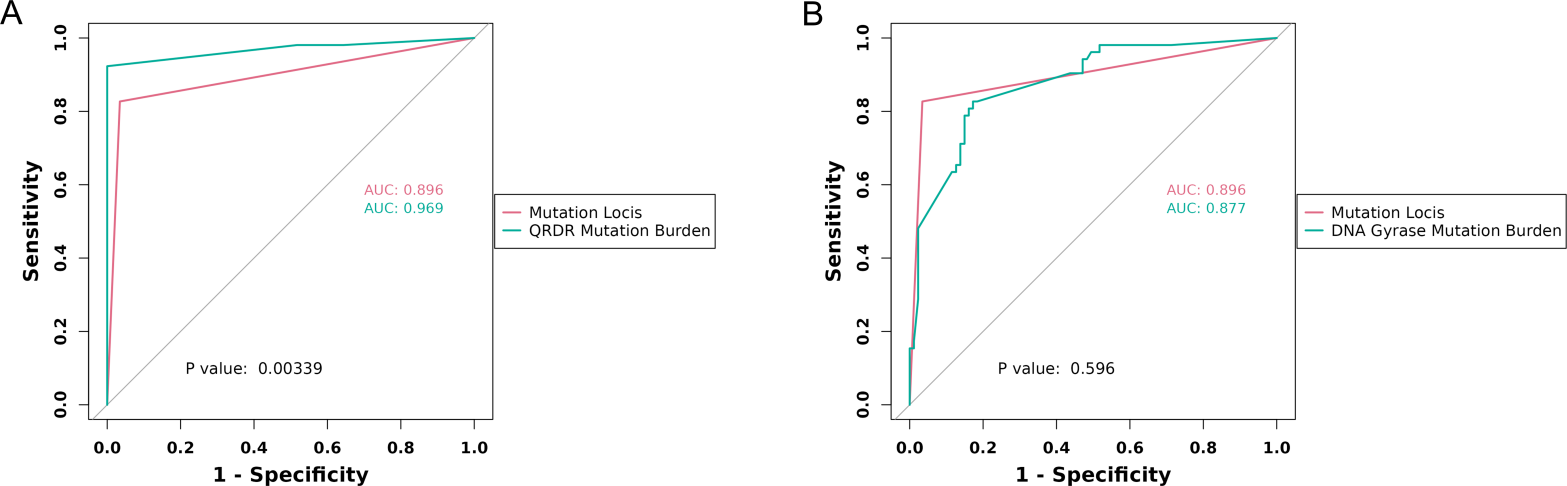
Comparison of the discriminatory ability of mutation loci and allele mutation frequency for FQ-resistant MTB isolates. (**A**) Comparison of the discriminatory ability of mutation loci and QRDR mutation burden. (**B**) Comparison of the discriminatory ability of mutation loci and DNA gyrase mutation burden.

We conducted a comparative assessment of the discriminative ability of mutation loci and mutation frequency burden in identifying FQ-resistant isolates, with detailed results depicted in Fig. 1. The outcomes revealed that in the diagnosis of FQ-resistant isolates, the AUC for the mutation frequency burden within the QRDR (0.969) exceeded that of the AUC for mutation loci (0.896), and this difference was statistically significant (*P* = 0.003). Conversely, there was no superior discriminative ability observed for FQ resistance when considering the target gene mutation frequency burden compared to mutation loci. These results signify that, in contrast to the 10 resistance loci, the mutation frequency burden within the QRDR exhibits a heightened capability to differentiate FQ-resistant MTB isolates.

### *In vitro* drug resistance testing of novel mutation sites

Previous studies have reported that the amino acid mutation S95T was initially suggested to be linked to FQ resistance. However, subsequent research has demonstrated that it represents a natural polymorphism in Beijing genotype strains (15–17). In our investigation, we identified the genotypes of all isolates, with 127 out of 139 isolates belonging to the Beijing genotype strains, 11 classified as non-Beijing genotype strains, and one isolate displayed mixed infection with both Beijing and non-Beijing genotypes. Consequently, in this study, we exclusively conducted *in vitro* validation to assess the correlation between the amino acid changes G512R, G520D, and G520T, located within the QRDR of the gyrB gene, and FQ resistance.

The control strain, pMV361-gyrB, exhibited a minimum inhibitory concentration (MIC) value of 0.13 mg/L under Lfx pressure. Conversely, the recombinant strains pMV361-gyrB-G512R, pMV361-gyrB-G520D, and pMV361-gyrB-G520T displayed MIC values of 0.13 mg/L, 0.25 mg/L, and 0.25 mg/L, respectively (specific results can be found in Fig. S5). These findings indicate that the amino acid mutations G520D and G520T are associated with FQ resistance.

### Comparative efficacy of expanded mutation loci and QRDR mutation frequency burden in diagnosing FQ-resistant isolates

Following expanding the resistant loci based on the *in vitro* drug susceptibility testing results, we identified a total of 11 mutation loci (12 amino acid mutations) in FQ-resistant isolates. Consequently, the sensitivity of diagnosing FQ-resistant MTB isolates increased from the original 82.69% to 90.38%.

**Fig 2:**
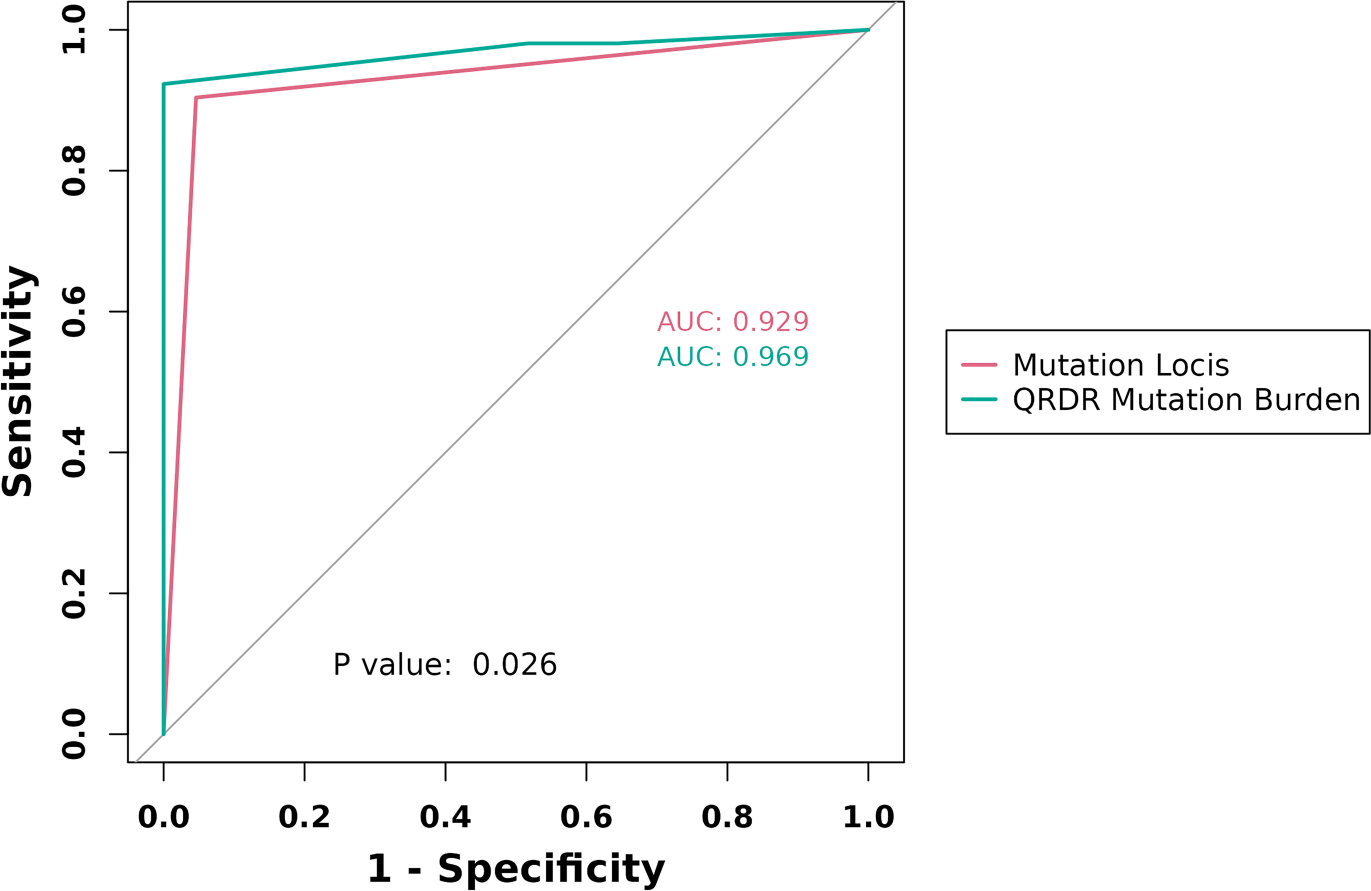
Comparison of the discriminatory ability of the expanded mutant loci and the QRDR mutation frequency burden for FQ-resistant MTB isolates.

Table 6 presents the comparative performance of expanded mutation loci and QRDR mutation frequency burden in diagnosing FQ-resistant MTB isolates. The QRDR mutation frequency burden exhibited superior sensitivity, specificity, DOR, Youden’s index, agreement rate, kappa value, PPV, and NPV when compared to mutation loci. The optimal diagnostic threshold for QRDR mutation frequency burden was determined to be 1.31%. The AUC of QRDR mutation frequency burden (0.969) remained higher than that of mutation loci (0.929) in diagnosing FQ-resistant isolates, with a statistically significant difference (*P* = 0.03) (Fig. 2). Furthermore, when considering the resistance mutation loci as the reference, setting the QRDR mutation frequency burden threshold at 1.31% improved the ability to accurately classify clinical MTB isolates as FQ-resistant by 3.60% (NRI = 3.60%), with statistical significance (*P* < 0.001).

**TABLE 6:** Evaluation of the efficacy of the mutation locus and QRDR mutation frequency burden in diagnosing FQ resistance in isolates

Type of mutation	Sensitivity (%)	Specificity (%)	DOR	Youden’s index	Agreement rate (%)	Kappa value	PPV (%)	NPV (%)	Threshold (%)
Mutation loci	90.38	95.40	195.05	0.86	93.53	0.86	92.16	94.32	
QRDR mutation frequency burden	92.31	100.00	***	0.92	97.12	0.94	100.00	95.60	1.31

## DISCUSSION

This study presents several key findings. First, it highlights a discrepancy in diagnostic sensitivity (82.7%) for FQ-resistant isolates when using mutation loci from the WHO drug resistance database, which differs from prior research (13, 18, 19). The results contribute valuable evidence to the field of evidence-based medicine. Second, our discovery of the *in vitro* association between specific amino acid mutations (G520D and G520T) improved the sensitivity without compromising the specificity, augmenting the existing mutation loci associated with FQ resistance. Lastly, we identified a QRDR allele frequency mutation detection threshold of 1.31%, which exhibited significantly higher sensitivity than that of widely available WGS analysis pipelines. This approach provides a more sensitive method for detecting FQ resistance, especially in cases with heterogeneous strains that may elude detection by many phenotypic and molecular assays (20). These results contribute robust data that provide support for establishing and updating rapid molecular diagnostic models for FQ-resistant MTB.

The nonsynonymous mutations observed in the MTB isolates encompassed various amino acid changes in the gyrA and gyrB genes, many of which have been previously associated with FQ resistance and are listed as such in the WHO resistance mutation database (8, 21, 22). While the S95T amino acid mutation has been linked to FQ resistance, its dependence on the MTB genotypic background has been demonstrated in previous research (15–17). Among the 139 analyzed isolates, the Beijing genotype strains constituted the majority (91.37%), with H37Rv chosen as the reference genome for the sake of generalization. Moreover, there were no statistically significant differences in the distribution of Beijing and non-Beijing genotype strains concerning FQ resistance (*P* = 0.49), and thus non-Beijing genotype strains were included in the analysis. Though the G512R amino acid mutation has been reported previously, it was only found to be statistically associated (23). This study is the first to report the direct association of G520D and G520T amino acid changes in the gyrB gene and FQ resistance.

While assessing MTB sensitivity to drugs based on amino acid changes at specific loci is a simple and convenient approach, we have observed instances where isolates exhibit low-frequency allele mutations at known drug resistance loci while still displaying a susceptible phenotype. This phenomenon, as observed in Huo’s study, with identifiable mutations in gyrA, including D94A, present in phenotypically susceptible MTB isolates according to the “gold standard” (24), may increase the false-positive rate of molecular diagnostic techniques relying solely on site-specific mutations. This highlights the importance of considering allele mutation frequency in our analysis.

To date, limited studies have explored the use of allele mutation frequency to diagnose FQ-resistant MTB. Ferrara Maruri *et al*. (13) found that dominant mutations in the gyrA gene could diagnose FQ resistance in 53 non-Beijing genotype clinical MTB isolates, but our results diverge significantly from theirs. In our study, the high allele mutation frequency primarily resulted from sequencing samples obtained from cultured MTB clinical isolates, leading to a relatively homogeneous population of dominant strains. Moreover, the extraordinary frequency of individual mutation loci and the low prevalence of isolates with simultaneous mutations at multiple resistance loci led to both mutation loci and dominant loci consistently contributing to the diagnosis of FQ-resistant isolates. Consequently, we chose to utilize the allele mutation frequency load in the QRDR region as the diagnostic indicator. After excluding strains with nonsynonymous mutations in the QRDR, the only remaining nonsynonymous mutation was the E21Q amino acid change located outside the QRDR region. Importantly, all corresponding strains were hypersensitive to FQs, underlying the rationale of selecting the QRDR region in our study.

There are a couple of limitations in this study: (1) tudy design—this case–control study design may have influenced diagnostic performance. Different study designs, such as cross-sectional or prospective cohort studies, could provide more robust evidence and (2) genetic diversity—the predominance of Beijing lineage strains among the isolates may limit the generalizability of the findings. Further research with diverse MTB lineages is needed to validate the results. These limitations should be considered when interpreting the study’s findings, and future research should explore different study designs and a broader range of MTB strains to enhance the generalizability and reliability.

In summary, while mutation loci diagnosis of FQ-resistant MTB remains a convenient approach, the potential for low-frequency allele mutations should be considered. The concept of QRDR allele mutation frequency burden offers an alternative perspective for diagnosing FQ-resistant MTB, though further research is needed to establish a standardized threshold for QRDR allele mutation frequency burden. Additionally, combining allele mutation frequency in WGS with mutation loci analysis presents a promising avenue for determining MTB drug resistance.

## SUPPLEMENTAL MATERIAL

Supplemental figures and tables spectrum.03930-23-s0001.docx Fig. S1-S5; Tables S1-S3.

ASM does not own the copyrights to Supplemental Material that may be linked to, or accessed through, an article. The authors have granted ASM a non-exclusive, world-wide license to publish the Supplemental Material files. Please contact the corresponding author directly for reuse.
