# Supplementary material for: Identification of novel resistance-associated mutations and discrimination within whole-genome sequences of fluoroquinolone-resistant Mycobacterium tuberculosis isolates
Source: Microbiol Spectr. 2024 Apr 30;12(6):e03930-23. doi: 10.1128/spectrum.03930-23 (PMC11237524; doi:10.1128/spectrum.03930-23)
Supplement: Supplemental figures and tables — Fig. S1-S5; Tables S1-S3. [file spectrum.03930-23-s0001.docx]

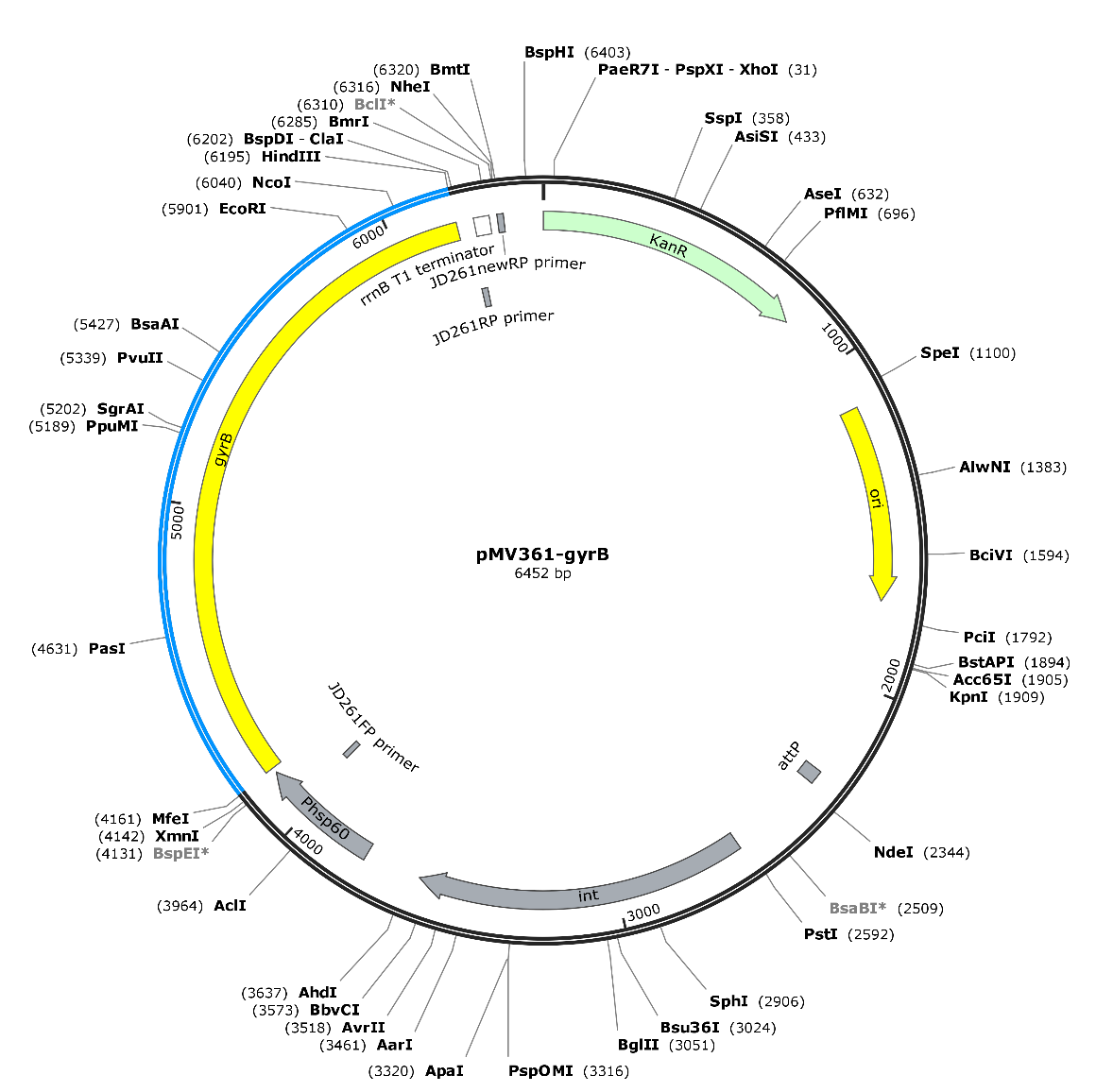


Figure S1 Example diagram of pMV361 plasmid ligated to gyrB gene


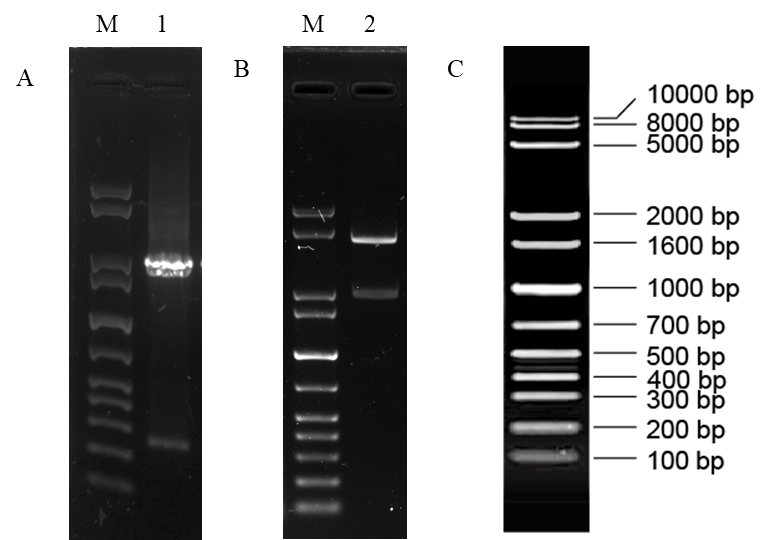


A is the PCR amplification electrophoresis of gyrB gene; B is the double digestion electrophoresis of pMV361-gyrB plasmid; C is the schematic diagram of DNA marker.

M is the marker, 1 and 2 are both corresponding target bands.

Figure S2 Amplification of gyrB gene and double digestion agarose electrophoresis of pMV361-gyrB plasmid


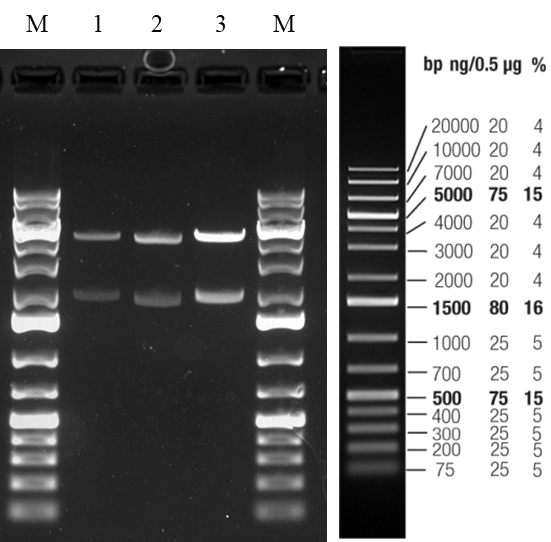


M is the marker; 1, 2 and 3 correspond to the constructed pMV361-gyrB-G512R plasmid, pMV361-gyrB-G520D plasmid and pMV361-gyrB-G520T plasmid

Figure S3 Double digestion agarose electrophoresis of mutant plasmids
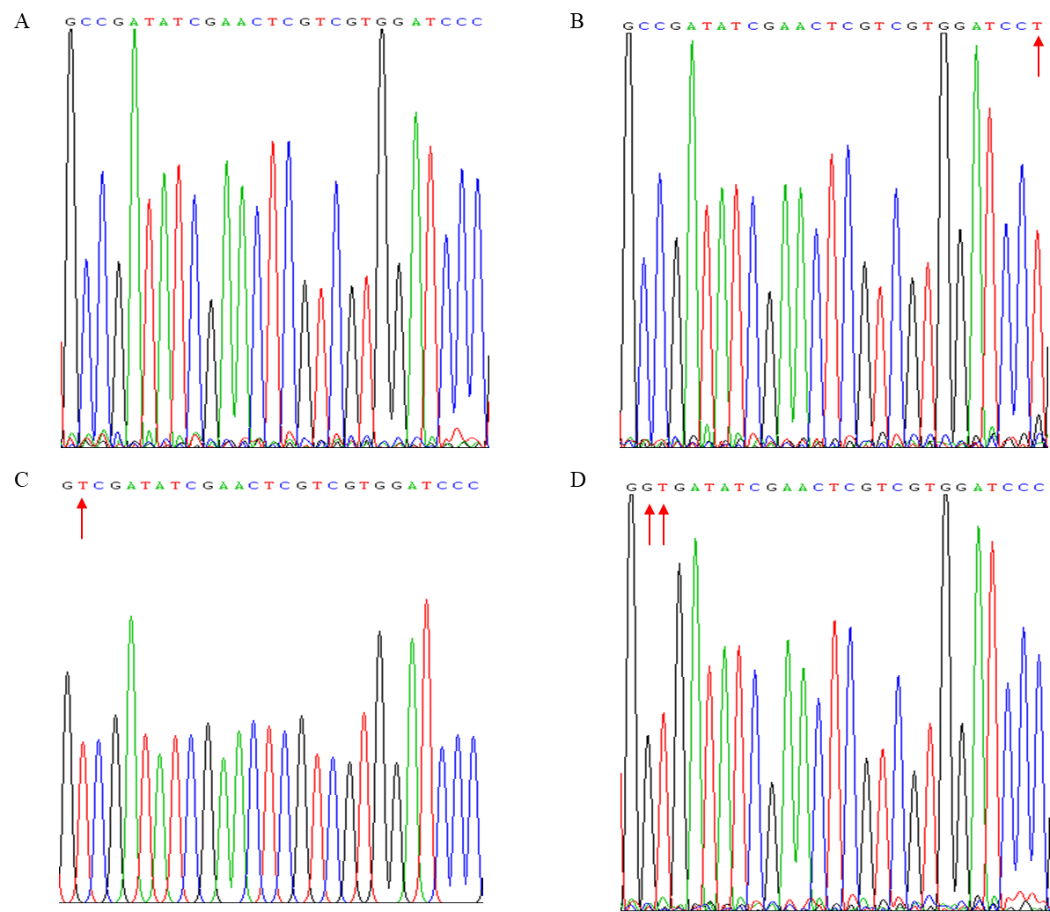


A is the sequenced fragment of pMV361-gyrB plasmid; B is the sequenced fragment of pMV361-gyrB-G512R plasmid; C is the sequenced fragment of pMV361-gyrB-G520D plasmid; D is the sequenced fragment of pMV361-gyrB-G520T plasmid

Figure S4 Local schematic diagram of mutation site sequencing


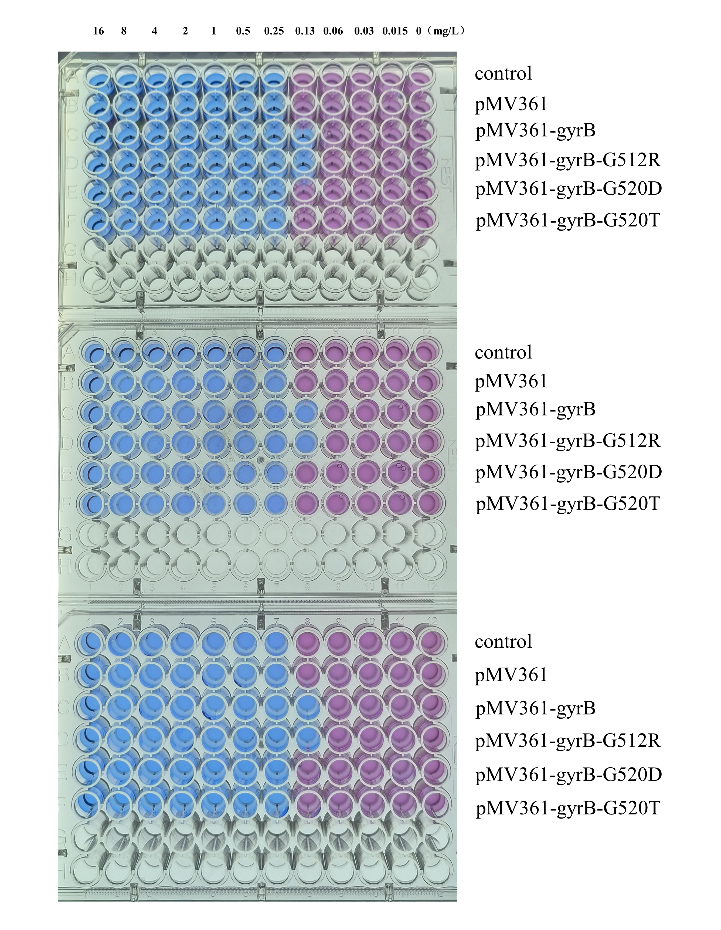


Figure S5 Graph of MIC value determination for the original and recombinant strains of mc^2^ 155

Table S1 Non-synonymous mutations in gyrA and gyrB genes outside QRDR Mutations

| Name | Gene | Condon Chang | Amino Acid Chang | Agreement rate between mutation and phenotypic drug sensitivity (n, %) |
| --- | --- | --- | --- | --- |
| gyrA | Rv0006 | Gcg>Tcg | A347S | 4（100.00） |
| gyrA | Rv0006 | Ctg>Atg | L361M | 4（100.00） |
| gyrA | Rv0006 | Tat>Cat | Y364H | 2（100.00） |
| gyrA | Rv0006 | gAC>gCG | D367A | 2（100.00） |
| gyrA | Rv0006 | cCa>cTa | A384V | 1（0.00） |
| gyrA | Rv0006 | Ggg>Cgg | G477R | 3（100.00） |
| gyrA | Rv0006 | cGc>cAc | R480H | 1（100.00） |
| gyrA | Rv0006 | ttG>ttC | L549F | 4（100.00） |
| gyrA | Rv0006 | GCg>CGg | A556R | 3（100.00） |
| gyrA | Rv0006 | tTg>tGg | L566W | 1（100.00） |
| gyrA | Rv0006 | tTc>tAc | F60Y | 2（100.00） |
| gyrA | Rv0006 | ACc>GAc | T618D | 4（100.00） |
| gyrA | Rv0006 | Tcc>Acc | S635T | 4（100.00） |
| gyrA | Rv0006 | Gtc>Atc | V651I | 4（100.00） |
| gyrA | Rv0006 | Gcc>Tcc | A667S | 4（100.00） |
| gyrA | Rv0006 | gGc>gAc | G668D | 39（64.10） |
| gyrA | Rv0006 | gaC>gaG | D669E | 4（100.00） |
| gyrA | Rv0006 | Cgc>Ggc | R789G | 1（100.00） |
| gyrA | Rv0006 | Ctc>Gtc | L12V | 2（100.00） |
| gyrA | Rv0006 | agG>agC | R143S | 1（100.00） |
| gyrA | Rv0006 | Gaa>Caa | E16Q | 3（100.00） |
| gyrA | Rv0006 | Gag>Cag | E21Q | 94（47.87） |
| gyrA | Rv0006 | aCc>aGc | T272S | 1（100.00） |
| gyrA | Rv0006 | Gtc>Atc | V291I | 1（100.00） |
| gyrA | Rv0006 | AtC>GtG | I316V | 4（100.00） |
| gyrA | Rv0006 | AtC>CtG | I328L | 4（100.00） |
| gyrB | Rv0005 | tCc>tTc | S447F* | 1（0.00） |
| gyrB | Rv0005 | CGc>AAc | R335N | 3（100.00） |
| gyrB | Rv0005 | AGc>GCc | S363A | 1（0.00） |
| gyrB | Rv0005 | gaA>gaT | E364D | 1（0.00） |
| gyrB | Rv0005 | Tcc>Gcc | S447A | 2（100.00） |
| gyrB | Rv0005 | Gcg>Tcg | A53S | 1（100.00） |
| gyrB | Rv0005 | ttG>ttC | L548F | 1（100.00） |
| gyrB | Rv0005 | gaG>gaC | E592D | 3（100.00） |
| gyrB | Rv0005 | cTg>cAg | L595Q | 3（100.00） |
| gyrB | Rv0005 | gaA>gaC | E604D | 4（100.00） |
| gyrB | Rv0005 | Tcg>Acg | S629T | 3（100.00） |
| gyrB | Rv0005 | Gtg>Atg | V258M | 1（0.00） |
| gyrB | Rv0005 | Acc>Ccc | T283P | 2（100.00） |
| gyrB | Rv0005 | atG>atC | M291I | 1（0.00） |
| gyrB | Rv0005 | gCc>gGc | A295G | 4（100.00） |
| gyrB | Rv0005 | CAc>ATc | H311I | 1（0.00） |

*Mutation loci associated with resistance to FQs in the WHO drug resistance database

Table S2 Detailed information on novel amino acid mutations within QRDR

| Original strain number | Novel Amino Acid Mutation | MIC value | Results Interpretation | Other mutations within QRDR | Genotyping |
| --- | --- | --- | --- | --- | --- |
| 131 | G512R | 2 | R | D94G, S95T | lineage2 |
| 163 | G512R | 4 | R | D94G, S95T | lineage2 |
| 437 | G512R | 2 | R | S95T | lineage2 |
| 923 | G512R | 2 | R | S95T | lineage2 |
| 197 | G520D | 1 | S | --- | lineage2 |
| 50 | G520T | 4 | R | S95T | lineage2 |
| 114 | G520T | 4 | R | S95T | lineage2 |
| 116 | G520T | 2 | R | S95T | lineage2 |
| 141 | G520T | 2 | R | S95T | lineage4 |

R indicates resistant; S indicates sensitive

Table S3 Cumulative allele mutation frequency burden of gyrA and gyrB genes

| Original number | DST | QRDR mutation frequency burden | DNA gyrase mutation frequency burden |
| --- | --- | --- | --- |
| 15 | S | 0.00 | 1.00 |
| 26 | S | 0.00 | 3.00 |
| 34 | S | 0.40 | 2.40 |
| 44 | R | 0.00 | 0.00 |
| 50 | R | 0.00 | 25.94 |
| 51 | R | 1.00 | 5.00 |
| 53 | R | 0.87 | 3.87 |
| 62 | R | 0.99 | 3.99 |
| 65 | R | 0.98 | 2.98 |
| 80 | R | 1.00 | 4.00 |
| 88 | R | 1.00 | 4.00 |
| 111 | S | 0.00 | 0.00 |
| 114 | R | 0.00 | 23.95 |
| 116 | R | 0.00 | 18.97 |
| 117 | R | 1.00 | 4.00 |
| 130 | S | 0.00 | 2.00 |
| 131 | R | 1.00 | 5.00 |
| 135 | S | 0.20 | 2.20 |
| 141 | R | 0.00 | 30.65 |
| 142 | S | 0.00 | 0.00 |
| 147 | S | 0.00 | 2.00 |
| 151 | R | 0.99 | 3.99 |
| 161 | R | 0.94 | 3.94 |
| 163 | R | 1.00 | 5.00 |
| 173 | S | 0.00 | 3.00 |
| 175 | S | 0.00 | 0.00 |
| 186 | R | 0.99 | 3.99 |
| 194 | S | 0.00 | 3.00 |
| 197 | S | 0.00 | 4.00 |
| 203 | S | 0.00 | 3.00 |
| 220 | R | 0.92 | 3.92 |
| 229 | R | 1.07 | 4.07 |
| 239 | S | 0.00 | 2.00 |
| 262 | S | 0.00 | 1.00 |
| 263 | S | 0.00 | 2.92 |
| 266 | R | 1.00 | 4.00 |
| 276 | S | 0.00 | 2.99 |
| 283 | S | 0.00 | 2.00 |
| 294 | R | 0.99 | 3.99 |
| 309 | R | 0.99 | 3.99 |
| 319 | S | 0.00 | 2.00 |
| 320 | S | 0.00 | 3.00 |
| 326 | S | 0.00 | 3.00 |
| 349 | R | 0.56 | 3.56 |
| 355 | S | 0.00 | 2.00 |
| 359 | R | 1.00 | 2.00 |
| 361 | R | 1.00 | 4.00 |
| 365 | S | 0.00 | 2.00 |
| 381 | R | 0.97 | 2.97 |
| 394 | R | 1.00 | 3.00 |
| 396 | S | 0.00 | 2.00 |
| 408 | S | 0.00 | 5.00 |
| 410 | S | 0.00 | 1.00 |
| 425 | R | 1.00 | 3.00 |
| 426 | S | 0.00 | 1.00 |
| 429 | R | 1.00 | 3.00 |
| 432 | S | 0.00 | 2.00 |
| 434 | S | 0.00 | 1.00 |
| 437 | R | 0.00 | 2.00 |
| 440 | S | 0.00 | 0.00 |
| 457 | R | 1.00 | 4.00 |
| 458 | S | 0.00 | 2.00 |
| 460 | S | 0.00 | 2.00 |
| 462 | S | 0.00 | 1.99 |
| 468 | R | 1.00 | 2.00 |
| 479 | S | 0.00 | 1.00 |
| 486 | R | 1.00 | 5.00 |
| 490 | S | 0.00 | 0.00 |
| 516 | S | 0.00 | 2.00 |
| 528 | R | 0.00 | 2.00 |
| 538 | S | 0.00 | 0.00 |
| 539 | R | 0.83 | 2.83 |
| 542 | R | 0.31 | 2.31 |
| 551 | R | 1.00 | 3.00 |
| 568 | S | 0.00 | 0.00 |
| 572 | S | 0.00 | 1.00 |
| 609 | R | 0.43 | 3.43 |
| 612 | R | 0.99 | 2.99 |
| 619 | S | 0.00 | 1.00 |
| 626 | S | 0.00 | 0.00 |
| 655 | S | 0.00 | 2.00 |
| 656 | S | 0.00 | 2.00 |
| 659 | S | 0.00 | 0.00 |
| 685 | R | 0.00 | 3.00 |
| 698 | R | 1.00 | 3.00 |
| 700 | R | 1.00 | 3.00 |
| 701 | S | 0.00 | 2.00 |
| 709 | R | 1.00 | 3.00 |
| 710 | R | 1.00 | 3.00 |
| 714 | S | 0.00 | 2.00 |
| 717 | S | 0.00 | 2.00 |
| 726 | S | 0.00 | 2.00 |
| 746 | S | 0.00 | 0.00 |
| 747 | S | 0.00 | 0.00 |
| 755 | R | 1.00 | 3.00 |
| 760 | R | 1.00 | 2.00 |
| 762 | S | 0.00 | 1.00 |
| 768 | S | 0.00 | 2.00 |
| 770 | S | 0.00 | 0.00 |
| 776 | S | 0.00 | 0.64 |
| 782 | R | 2.00 | 2.00 |
| 794 | S | 0.00 | 2.00 |
| 803 | S | 0.00 | 0.00 |
| 812 | S | 0.00 | 2.00 |
| 835 | R | 1.00 | 3.00 |
| 836 | S | 0.00 | 3.00 |
| 842 | S | 0.00 | 1.00 |
| 848 | S | 0.00 | 0.00 |
| 863 | R | 1.00 | 3.00 |
| 870 | S | 0.00 | 3.00 |
| 878 | S | 0.00 | 3.00 |
| 884 | S | 0.00 | 0.00 |
| 914 | S | 0.00 | 2.00 |
| 916 | S | 0.00 | 0.00 |
| 918 | S | 0.00 | 1.00 |
| 922 | S | 0.00 | 0.00 |
| 923 | R | 0.00 | 2.00 |
| 926 | S | 0.00 | 0.00 |
| 927 | S | 0.00 | 0.00 |
| 928 | S | 0.00 | 2.00 |
| 929 | S | 0.00 | 0.00 |
| 930 | S | 0.00 | 0.00 |
| 934 | R | 0.94 | 1.94 |
| 938 | S | 0.00 | 1.99 |
| 940 | S | 0.00 | 1.00 |
| 941 | S | 0.00 | 1.00 |
| 945 | S | 0.00 | 2.00 |
| 946 | S | 0.00 | 2.00 |
| 947 | S | 0.00 | 1.00 |
| 948 | S | 0.00 | 0.00 |
| 950 | S | 0.00 | 2.00 |
| 951 | S | 0.00 | 1.00 |
| 953 | S | 0.00 | 0.00 |
| 954 | S | 0.00 | 1.00 |
| 958 | S | 0.00 | 0.00 |
| 962 | S | 0.00 | 2.00 |
| 965 | S | 0.00 | 3.00 |
| 969 | R | 0.99 | 2.99 |
| 974 | S | 0.00 | 0.00 |

R indicates resistant; S indicates sensitive
